# Short-term results after laparoscopic repair of giant hiatal hernias with pledgeted sutures: a retrospective analysis

**DOI:** 10.1007/s10029-019-01890-3

**Published:** 2019-01-25

**Authors:** M. Weitzendorfer, R. Pfandner, S. A. Antoniou, C. Schwaiger-Hengstschläger, K. Emmanuel, O. O. Koch

**Affiliations:** 1 Department of Surgery, Paracelsus Medical University, Müllner Hauptstraße 48, 5020 Salzburg, Austria; 2 Department of General and Visceral Surgery, Ordensklinikum Linz Barmherzige Schwestern, Linz, Austria; 3 Department of Colorectal Surgery, Royal Devon and Exeter NHS Foundation Trust, Exeter, UK

**Keywords:** Antireflux surgery, Hiatal closure, GERD, Pledgets, Hiatal hernia

## Abstract

**Purpose:**

This study investigates if pledgeted sutures for hiatal closure could be an alternative to mesh for the surgical treatment of large hiatal hernia.

**Methods:**

Forty-one patients who underwent laparoscopic 270° Toupet fundoplication with pledgeted sutured crura between September 2014 and April 2017 were evaluated with regard to recurrence of hiatal hernia at 3 months and 1 year after surgery. Indication for pledgets was a hiatal surface area of at least 5.60 cm^2^, or migration of more than 1/3 of the stomach into the thorax or preoperative hernia size > 5 cm. The integrity of repair was assessed using a barium swallow test 3 months and 1 year after surgery.

**Results:**

All operations could be completed laparoscopically with no intraoperative complications. Until study end no complications related to the pledgets have occurred. Forty-four of 50 patients (88.0%) completed the follow-up radiographic examination 3 months (mean 12.7 weeks) after surgery, and 37 patients (74.0%; mean 55.1 weeks) 1 year after surgery. Postoperative recurrence was diagnosed in 3/44 patients (6.8%) at 3 months, and in 4/37 patients (10.8%) at 1 year follow-up. Only one patient was symptomatic, 1 year after surgery (2.7%). All other patients with reherniations were asymptomatic at time of the study.

**Conclusions:**

Utilization of pledgets to reinforce hiatal sutures seems safe and shows a quite low early recurrence rate compared to other methods. Long-term data will allow firm conclusions as to whether pledgeted sutures are an appropriate solution for the treatment of giant hiatal hernias.

## Introduction

Several studies have proven that laparoscopic antireflux surgery (LARS) is safe and effective with excellent long-term functional outcomes for the treatment of gastroesophageal reflux disease (GERD) [1], whereas reports of laparoscopic repair of giant and paraesophageal hernias have been disappointing with short-term recurrence rates of more than 42% [2, 3]. Therefore, many authors recommend using a mesh for hiatal closure to reduce the rate of recurrence in giant hiatal hernia repair. However, possible complications due to the mesh have to be taken into account [4]. The most feared complication after mesh-hiatoplasty is erosion and migration of the mesh. Several papers report on mesh-associated complications, such as infection, stricture, erosion, and dysphagia. These complications have to be feared with all types of mesh, even after using biological mesh [5, 6]. Stadlhuber et al. report from partly severe complications due to mesh including esophagectomies and gastrectomies [5].

Because of these possible complications, many surgeons are reluctant to use a mesh. Another reason discouraging the use of mesh is the costs, especially in times of a every day growing cost pressure. An alternative cost-effective method without the risks of mesh-related complications, but with acceptable recurrence rates, following a giant hiatal hernia surgery would be desirable. For some authors this alternative could be the usage of pledgets for primary crural closure [7, 8].

Since there are scarce data on the outcome of reinforced crural repair using pledgets, the purpose of this study was to evaluate the outcome of patients undergoing giant hiatal hernia repair with pledgeted sutures under standardised conditions in a single institution.

## Materials and methods

From our prospective database we retrospectively analysed the results from 50 consecutive patients. Since October 2014 we have used PTFE-pledgets to reinforce hiatal sutures during primary surgery in the presence of a large hiatal hernia. We defined large as migration of more than 1/3 of the stomach into the thorax or preoperative hernia size > 5 cm. Furthermore using the formula developed by Granderath et al. the hiatal surface area (HSA) was calculated during surgery and a large hiatal hernia was defined as a defect of more than 5.60 cm^2^ [9]. Until April 2017, 50 patients underwent laparoscopic 270° Toupet fundoplication with pledgeted sutured crura.

All patients receiving LARS at our department were invited to undergo radiographic examination 3 months and 1 year after surgery. Preoperatively an evaluation of symptoms, esophagogastroduodenoscopy and barium swallow test (cinematographic X-ray film) was performed. HR-manometry and 24 h-pH-metry-impedance-measurment were used selectively, especially to rule out high-grade motility disorders, since indication for surgery was symptoms resulting from a large hiatal hernia.

### Surgical technique

In a recent publication we have described our technique of laparoscopic fundoplication/hiatoplasty in detail [10]. We seek to preserve the crural integrity, to completely resect the sac and gastroesophageal fat-pad dissection is performed routinely.

**Fig. 1:**
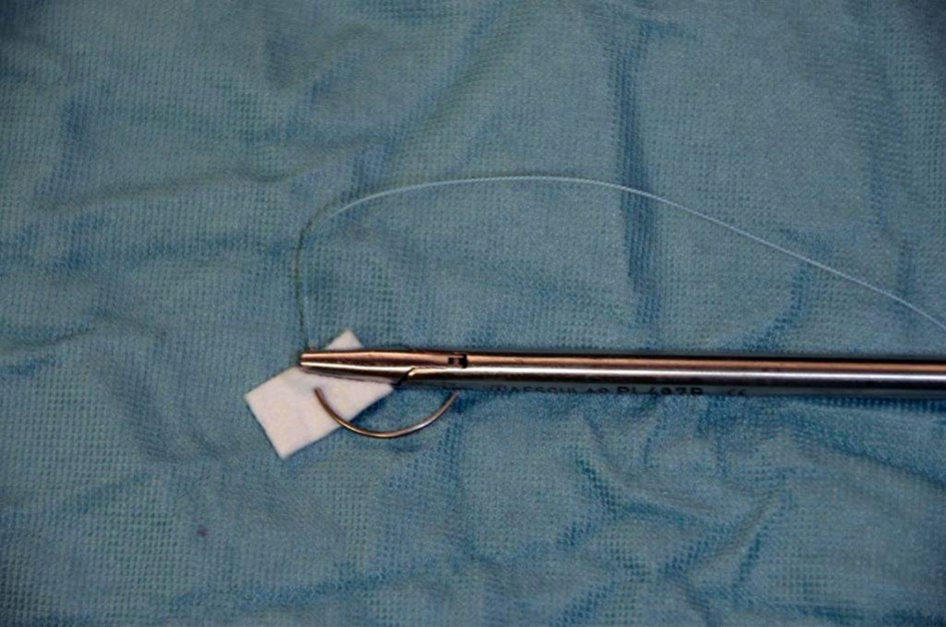
Prepared needle with pledget to insert over trocar

**Fig. 2:**
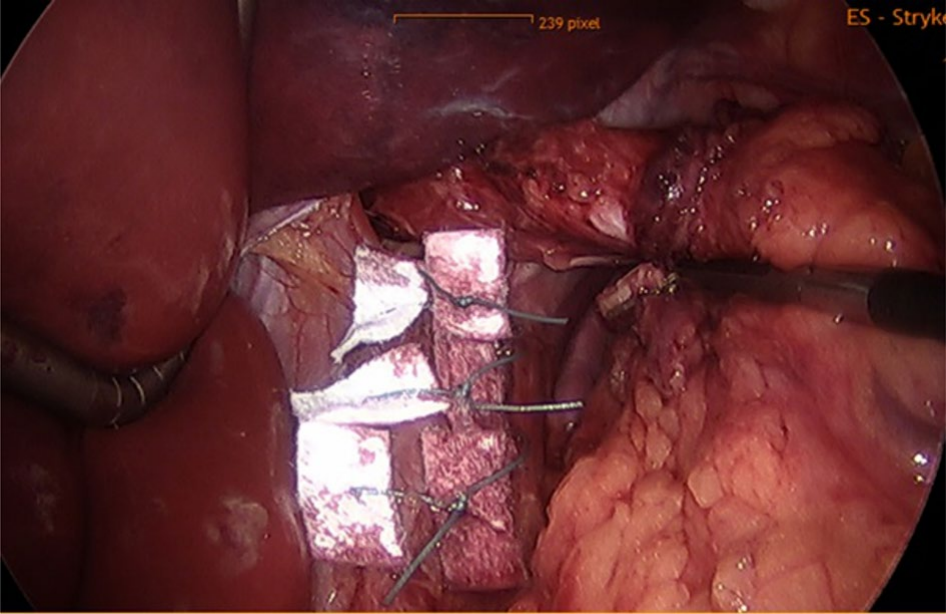
Hiatus closed with interrupted pledgeted no. 0 non absorbable sutures

After exact dissection of the right and left crus and the crural commissure, we measured the hiatal defect. In all patients the hiatal crura were closed using interrupted pledgeted 0 non absorbale ethibond sutures (Figs. 1, 2). On average 4–5 sutures were needed with two pledgets each. Standardized polytetrafluoroethylene (PTFE) pledgets measuring 15 × 10 × 1.6 mm (Santec GmbH, 63868 Grosswallstadt, Germany) were used. After closing the crura, a 270° Toupet fundoplication was fashioned in all patients.

### Barium X-ray

**Fig. 3:**
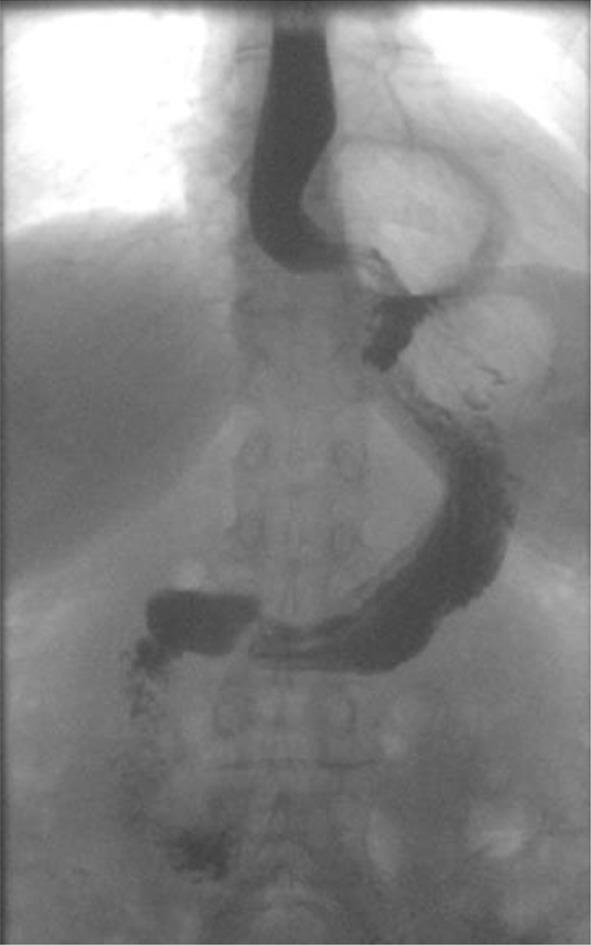
Barium X-ray film demonstrating reherniation

A single radiologist assessed the integrity of repair using a barium swallow test (cinematographic X-ray film). The video esophagrams were performed according to a protocol and all views (anteroposterior and oblique) were obtained in upright and supine positions. Every patient had five swallows of liquid barium using the same amount of liquid. Cinematographies were performed at 3 months and 1 year after operation. Anatomic recurrence was defined as any evidence of herniation of gastric tissue above the level of diaphragm (Fig. 3).

### Statistical analysis

Statistical analysis was performed using the Statistical Product and Service Solutions (SPSS) computer program (SPSS Inc., Chicago, IL, USA). Data are presented as means ± standard deviation, range, or percentage.

## Results

The mean age of the patients at time of the operation was 67 years (min. 31–max. 85 years). There were 33 female and 17 male patients, with a mean Body Mass Index (BMI) of 27.93 (min. 20.96–max. 40.04). The mean size of the HSA was 7.13 cm^2^ (5.85–21.10 cm^2^).

All procedures could be completed laparoscopically with no intraoperative or postoperative complications, except for one postoperative pneumonia. The patient had to undergo antibiotic therapy. After 1 week recovery was complete. A Collis gastroplasty was performed in two patients because of short esophagus.

Forty-four of 50 patients completed the follow-up radiographic examination (88.0%) at 3 months (mean 12.7 weeks), and 37 patients (74.0%; mean 55.1 weeks) at 1- year follow-up. All patients who did not appear in person for follow-up were contacted. Reason for loss of follow-up was in all cases that patients refused to further participate due to long-distance constraints.

Postoperative recurrence (recurrent hiatal hernia and reflux) was diagnosed in 3/44 patients (6.8%) at 3 months, and in 4/37 (10.8%) at 1-year follow-up. One patient with recurrent hiatal hernia was symptomatic 3 months after surgery; he suffered from dysphagia and GERD symptoms, which was evaluated by symptom questioning. This patient underwent the only redo surgery so far. Disrupted sutures were found intraoperatively and considered as reason for failure.

A laparoscopic Collis gastroplasty was performed due to short esophagus with an uneventful postoperative course. Currently the patient is asymptomatic and without reherniation.

One year after surgery recurrence rate was 10.8% but only one patient (2.7%) was symptomatic. All other patients with a recurrent hiatal hernia were asymptomatic at time of the examination. Until completion of the study, no pledget related complications have occurred.

## Discussion

Laparoscopic repair of large hiatal hernias is associated with a high incidence of postoperative reherniation [2]. To improve the recurrence rate, surgeons started to use a mesh to reinforce the hiatal closure. In 1999 Carlson et al. reported the results of the first randomized trial comparing sutures with mesh showing good early outcome [11]. Granderath et al. analysed 100 patients undergoing laparoscopic Nissen fundoplication with either simple suture cruroplasty or nonabsorbable polypropylene (PP) mesh placement. One year after surgery the mesh group showed significantly less hernia recurrences [12]. In short-term follow-up Oelschlager et al. reported a lower recurrence rate for biologic mesh compared to sutures (9% versus 24%) [13].

However, in long-term follow-up both had a dissatisfying high recurrence rate with 59% in the suture cruroplasty group, and 54% in the biologic prosthetic group [14]. The most recent trial on the issue is the study of Watson et al., where the authors compared three methods of repair: sutures versus absorbable mesh versus nonabsorbable mesh. Although the differences between the three different methods were not significant, it is worth emphasising that the group with non-absorbable mesh was associated with the lowest recurrence rate of 12.8% after 6 months compared to 23.1% after suture repair and 30.8% after repair with absorbable mesh [15].

In our study we used pledgets as an alternative to mesh and in 10.8% of the patients a recurrent hernia was diagnosed on barium X-ray at 1 year after surgery. Compared to the data of the above mentioned study this seems a satisfactory outcome, with a comparable number of patients and follow-up. However, a limitation of this study is that 1-year follow-up is too short to come to firm conclusions, but we believe that is also important to report early data since the moment of reherniation after surgery is another topic of interest.

A recent meta-analysis on the basis of the four existing RCT found comparable effect sizes for recurrence of hiatal hernia or wrap migration (OR 2.01, 95% CI 0.92, 4.39, *P* = 0.07). Nevertheless, the pooled data on reoperation in the meta-analysis showed a significant higher risk of revisional surgery after suture cruroplasty compared with prosthetic hiatal herniorrhaphy (OR 3.79, 95% CI 1.20, 11.99, *P* = 0.02) [16].

Therefore, a standard indication for the use of prosthetic mesh for hiatal closure still does not exist at this time. Furthermore, it is not clear which type of mesh, particularly which size and shape to use [17]. Müller-Stich et al. performed an experimental study with porcine models comparing three types of mesh. They circularly placed PP, polyester (PET) or PTFE meshes at the esophageal hiatus, and they found that PP-meshes demonstrated the most appropriate characteristics for augmentation at the hiatus [18].

Recently, we could demonstrate that reherniation is more likely the larger the hiatal defect is. Thus, we believe that the indication of using prosthetic material to buttress the hiatal closure should be dictated by the size of the hiatal defect and not on an individualized basis [10]. Unfortunately, so far no standard method to measure the size of the hiatus during surgery has been established and previous studies did not state the size of the hiatus. Therefore, comparisons with previous studies are not possible.

Some authors propose that the number of sutures used at primary crural closure correlates with the size of the hiatal defect and can be used as an indirect gauge of hiatal defect size [7]. We believe that to come to firm conclusions in future studies that compare different methods of hiatoplasty, the size of the hiatal defect or at least the number of sutures should be stated.

Several authors recommend to use prosthetic material not only in case of a all large hiatal hernia, but also in patients in whom the crura seems weak or damaged [19]. The usage of pledgets could be of advantage, especially in patients with weak or damaged crura since they should prevent the sutures from cutting through tissue. Since the esophageal hiatus is a very dynamic area with constant movements of the diaphragm, the esophagus, the stomach, and the pericardium, a gradual “sawing”-effect of the sutures through the crura is conceivable.

Unfortunately, data of the quality of the crura of the patients in this study were not available and, therefore, we cannot make an accurate assertion.

The fact that the esophageal hiatus is such a dynamic area and the possible direct contact of organs to the mesh explains the possible complications like local erosion or migration of the mesh into the oesophagus or stomach [5].

Since pledgets are an artificial material, complications due to pledgets can be expected. Several groups have used pledgets to buttress the wrap in the past. Dally et al. retrospectively assessed complications related to pledgets after fundoplication in their department. They identified 11 patients of 1175 fundoplications who had symptomatic pledget erosion, which led them to abandon the technique. Similar problems associated with erosion and migration of Teflon prostheses were described [20]. However, these complications occurred when pledegets were used to buttress the wrap. We used pledgets to buttress the hiatal closure, and so far no complications due to pledgets have occurred. One patient with symptomatic recurrent hernia underwent redo surgery. At surgery, adhesions between pledgets and tissue could be found, but no erosion or migration. To prevent a reherniation and because a short esophagus was suspected, a laparoscopic Collis gastroplasty was performed without complications. Currently, the patient is asymptomatic and without evidence of hernia recurrence.

In times of intense economic considerations, the cost of the prosthetic material is an important issue. One package with six pledgets used in this study costs 13.22€, whereas for hiatoplasty a maximum of two packages were necessary, which makes a maximum price of 26.44€. The commonly used kind of absorbable mesh for hiatal closure, like the Symbotex™ Composite Mesh (Medtronic, Minneapolis, Minnesota, USA) or GORE^®^ BIO-A^®^ Tissue Reinforcement (W. L. Gore & Associates, Newark, Delaware, USA), cost around 450€. The price of the nonabsorbable TiSURE^®^ mesh (pfm medical GmbH, Köln, Germany) is about 275€. There is thus an obvious cost benefit of pledgeted sutures compared to mesh repair. A prospective trial comparing mesh versus pledgets would be interesting. If long-term outcome is similar using pledgets, they could be regarded as a cost-effective alternative.

In summary, the short-term results of this study suggest that pledgets could be at least an option for hiatoplasty, since the use of pledgets to reinforce hiatal sutures seems safe and shows a quite low early recurrence rate compared to other methods. Long-term data deriving from randomized trials comparing pledgets with mesh will allow drawing firm conclusions.
